# Dual RNA-seq in filarial nematodes and *Wolbachia* endosymbionts using RNase H based ribosomal RNA depletion

**DOI:** 10.3389/fmicb.2024.1418032

**Published:** 2024-05-20

**Authors:** Lindsey J. Cantin, Vanessa Gregory, Laura N. Blum, Jeremy M. Foster

**Affiliations:** ^1^Biochemistry and Microbiology Division, New England BioLabs, Ipswich, MA, United States; ^2^Applications and Product Development, New England BioLabs, Ipswich, MA, United States

**Keywords:** filariasis, *Wolbachia*, dual RNA sequencing, symbiosis, RNA enrichment, ribosomal depletion

## Abstract

Lymphatic filariasis is caused by parasitic nematodes and is a leading cause of disability worldwide. Many filarial worms contain the bacterium *Wolbachia* as an obligate endosymbiont. RNA sequencing is a common technique used to study their molecular relationships and to identify potential drug targets against the nematode and bacteria. Ribosomal RNA (rRNA) is the most abundant RNA species, accounting for 80–90% of the RNA in a sample. To reduce sequencing costs, it is necessary to remove ribosomal reads through poly-A enrichment or ribosomal depletion. Bacterial RNA does not contain a poly-A tail, making it difficult to sequence both the nematode and *Wolbachia* from the same library preparation using standard poly-A selection. Ribosomal depletion can utilize species-specific oligonucleotide probes to remove rRNA through pull-down or degradation methods. While species-specific probes are commercially available for many commonly studied model organisms, there are currently limited depletion options for filarial parasites. Here, we performed total RNA sequencing from *Brugia malayi* containing the *Wolbachia* symbiont (*w*Bm) and designed ssDNA depletion probes against their rRNA sequences. We compared the total RNA library to poly-A enriched, Terminator 5′-Phosphate-Dependent Exonuclease treated, NEBNext Human/Bacteria rRNA depleted and our custom nematode probe depleted libraries. The custom nematode depletion library had the lowest percentage of ribosomal reads across all methods, with a 300-fold decrease in rRNA when compared to the total RNA library. The nematode depletion libraries also contained the highest percentage of *Wolbachia* mRNA reads, resulting in a 16–1,000-fold increase in bacterial reads compared to the other enrichment and depletion methods. Finally, we found that the *Brugia malayi* depletion probes can remove rRNA from the filarial worm *Dirofilaria immitis* and the majority of rRNA from the more distantly related free living nematode *Caenorhabditis elegans*. These custom filarial probes will allow for future dual RNA-seq experiments between nematodes and their bacterial symbionts from a single sequencing library.

## 1 Introduction

Filariasis is an infectious disease caused by parasitic nematodes. The disease is one of the leading causes of disability worldwide, with an estimated 2 billion people at risk of infection in the tropical and sub-tropical regions of Africa, Asia, and South America (Taylor et al., 2010; Bhalla et al., 2013). Filarial parasites are transmitted through blood feeding insect vectors and can infect both humans and animals (Chandy et al., 2011). The pathology of the disease, from host preference to disease outcomes, is determined by the filarial species present. Lymphatic filariasis can lead to elephantiasis and disfigurement, which is caused by *Brugia malayi* (*B. malayi*) and *Wuchereria bancrofti* infections in humans (World Health Organization, 2019; Medeiros et al., 2021). Subcutaneous filariasis is caused by *Loa loa, Onchocerca volvulus*, and *Mansonella streptocerca* infections and can lead to skin issues and blindness (WHO Expert Committee on Onchocerciasis Control, 1993; Boussinesq, 2006; Mediannikov and Ranque, 2018). *Dirofilaria immitis* (*D. immitis*), also known as heartworm, is a filarial nematode that primarily infects canid species, leading to lung and heart damage (Simón et al., 2012; Anvari et al., 2020). Combinations of albendazole, ivermectin, and diethylcarbamazine are used to control the spread of the disease by targeting microfilariae (Campbell, 1991; Richards et al., 2001; Mackenzie et al., 2002; Molyneux et al., 2003). However, drugs that kill adult stages are necessary to treat active infections, as the worms can survive long-term in the vertebrate hosts.

The majority of filarial nematodes contain *Wolbachia* as an obligate intracellular bacterial symbiont at all stages of their life cycle (Mclaren et al., 1975; Sironi et al., 1995; Bandi et al., 1998, 2001; Quek et al., 2022). *Wolbachia* are critical to the development, reproduction, and long-term survival of adult nematodes, most likely by providing metabolites from biological pathways absent in the worms (Foster et al., 2005; Wu et al., 2009; Darby et al., 2012; Li and Carlow, 2012; Luck et al., 2016). However, the molecular basis for this symbiosis is still poorly understood. Anti-*Wolbachia* treatments with antibiotics, such as doxycycline and rifampicin, are a promising strategy for filariasis eradication by sterilizing and killing adult worms (Taylor et al., 2005, 2019; Bazzocchi et al., 2008; Hoerauf et al., 2008; Specht et al., 2008; Supali et al., 2008; Coulibaly et al., 2009; Mand et al., 2009; Wanji et al., 2009; Johnston et al., 2014, 2021; Aljayyoussi et al., 2017; Clare et al., 2019). Gene expression analysis for both the nematode and the bacteria during different life-cycle stages can help to identify the biological relationship between them and to determine putative drug targets.

RNA-sequencing is a powerful approach that can be used to identify and quantify genes expressed under certain conditions or throughout the life cycle (Wang et al., 2009; Hrdlickova et al., 2017). Ribosomal RNA (rRNA) makes up between 80 and 90% of total RNA in a cell (Zhao et al., 2018; Deng et al., 2022). Therefore, rRNA must be removed prior to sequencing to analyze the smaller fraction of messenger RNA (mRNA) and non-coding RNAs (ncRNA) involved in interesting cellular functions. Typically, rRNA is removed during library preparation using Poly(A) enrichment or ribosomal depletion methods (Sultan et al., 2014; Zhao et al., 2014; Schuierer et al., 2017). Poly(A) enrichment is commonly used when studying eukaryotes by selecting for RNA species containing a poly(A) tail, usually mRNA and some ncRNAs, while removing all others, including rRNA. However, poly(A) tails are mostly absent from bacterial RNAs, resulting in a loss of all bacterial signal from libraries prepared with this method (Prezza et al., 2020; Xiang et al., 2022).

Ribosomal depletion can be used to remove only the rRNA, allowing for eukaryotic mRNA, bacterial mRNA, and ncRNA to all be sequenced within the same library. Ribosomal depletion typically requires sequence specific probes for either the pulldown or targeted degradation of rRNA (Adiconis et al., 2013; Wahl et al., 2022). Commonly studied model organisms, such as mouse, rat, human and bacteria, have commercially available kits for ribosomal depletion. There are limited depletion options for filarial nematodes and other *Wolbachia* containing hosts, preventing dual RNA-sequencing of the hosts and bacterial endosymbionts together (Kumar et al., 2012, 2016; Grote et al., 2017; Chung et al., 2019). 5′ phosphate dependent exonucleases, such as Terminator and Xrn1, have also been used to selectively degrade large rRNA molecules containing a 5′ monophosphate (He et al., 2010; Kang et al., 2011; Grote et al., 2017; Wangsanuwat et al., 2020). However, this method does not remove the 5S rRNA and is greatly affected by the secondary structure of RNAs, lowering its efficiency and increasing off-target effects compared to probe-based depletion.

Here, we present ribosomal depletion probes for *B. malayi* and its *Wolbachia* endosymbiont, *w*Bm, to perform dual RNA-seq. We also provide a proof of principal for the updated publicly available NEBNext Custom RNA Depletion design tool (https://depletion-design.neb.com), incorporating a clustering step to remove redundancy in the probe set. We compared libraries prepared with our custom filarial nematode depletion probes and RNase H digestion to RNA-seq libraries prepared with no treatment (total RNA), poly(A) enrichment, 5′ phosphate dependent exonuclease digestion, and commercially available NEBNext rRNA depletion with a combination of probes designed for Human/Mouse/Rat and Bacteria. We find almost complete rRNA removal and an increase in *Wolbachia* mRNA reads with the *B. malayi* specific probes, improving upon results seen with the other methods mentioned above. The custom depletion probes were also tested for their ability to remove rRNA from total RNA of the closely related filarial nematode *D. immitis* and the distantly related free-living nematode *Caenorhabditis elegans* (*C. elegans*). We found an almost 70% reduction in rRNA reads in the *C. elegans* library and an over 99% reduction in the *D. immitis* library, showing that this custom probe design can be used as a pan-filariae ribosomal depletion method for dual RNA-seq with their *Wolbachia* endosymbionts.

## 2 Materials and methods

### 2.1 *B. malayi* tissue collection

Thirty live adult *B. malayi* females were received from the NIH-NIAID Research Reagent Resource Center (FR3, Athens, GA; Michalski et al., 2011). Immediately after arrival, the worms were placed at 37°C with 5% CO_2_ in freshly prepared RPMI 1640 media containing 10% heat inactivated fetal bovine serum (Thermo Fisher Scientific, Waltham, MA), 2 mM L-glutamine, 5 g/L glucose, 100 ug/mL streptomycin, 100 U/mL penicillin and 250 ng/mL amphotericin (Millipore Sigma, Burlington, MA). The worms were incubated overnight for a minimum of 16 h. The worms were sorted into groups of 3 and washed twice with 1X PBS. All washes were removed, and the worms were snap frozen in liquid nitrogen and stored in 1.5 mL LoBind tubes at −80°C.

### 2.2 *D. immitis* tissue collection

Five live adult female *D. immitis* were shipped overnight from the FR3. Upon arrival, the worms were rinsed two times in 1X PBS. All liquid was removed. The worms were placed individually in 1.5 mL DNA LoBind tubes and flash frozen in liquid nitrogen. The samples were stored at −80°C until future use.

### 2.3 *C. elegans* tissue collection

N2 strain *C. elegans* nematodes were maintained on 10 cm Nematode Growth Medium (NGM) agar plates seeded with OP50 *E. coli* (Chaudhuri et al., 2011). Once the plate was starved, the worms were recovered by rinsing the plates with 10 mL of M9 buffer (22 mM KH_2_PO_4_, 42 mM Na_2_HPO_4_, 86 mM NaCl) and transferring the liquid containing the worms to a 15 mL conical tube. The worms were pelleted by spinning at 400 × *g* for 5 min. To wash the worm pellet, the supernatant was removed and another 10 mL of M9 buffer was added to the tube to resuspend the pellet. The worms were washed a total of 5 times. After the final spin, 9 mL of the supernatant was discarded. The worm pellet was resuspended in the remaining buffer and transferred to a 1.5 mL DNA LoBind tube. The tube was spun at 400 × *g* for 5 min and the supernatant was removed. The *C. elegans* pellet was flash frozen in liquid nitrogen and stored at −80°C until future use.

### 2.4 Total RNA extraction, sequencing, and analysis

Total RNA was extracted separately from individual aliquots for each nematode species, including *B. malayi* (three adult female worms), *C. elegans* (worm pellet from a starved 10 cm plate, mixed sex), and *D. immitis* (one adult female worm). For each sample, 0.5 mL of TRIzol (Thermo Fisher Scientific, Waltham, MA) was added to the 1.5 mL LoBind tube containing the frozen tissue. The tissue and TRIzol mix were then moved to a sterile 2 mL Dounce Homogenizer. The nematodes were homogenized using 10 strokes of the A pestle and 10–20 strokes of the B pestle. The samples were incubated at room temperature for 5 min. We added 100 μL of chloroform to each sample and mixed by manually shaking for 10 s. After an additional 3-min incubation at room temperature, the aqueous and organic phases were separated by spinning at 12,000 × *g* for 15 min at 4°C. The upper clear aqueous phase containing the RNA was transferred to a clean 1.5 mL DNA LoBind tube. The volume in each tube was measured with a P1000 pipette and an equal volume of >95% ethanol was added. We used the Monarch Total RNA Miniprep Kit (New England BioLabs, Ipswich, MA) to complete the cleanup of the RNA, using the total RNA purification from TRIzol-extracted samples protocol as described, with the inclusion of the optional DNase I treatment. RNA quality was measured by running 1 μL of the purified RNA sample on a Bioanalyzer using the RNA 6000 pico kit (Agilent, Santa Clara, CA) with the Eukaryote Total RNA Pico Series II assay. All RNA used for further experiments had an RNA Integrity Number (RIN) of 8 or above.

Total RNA-seq libraries for each species were prepared using the NEBNext Ultra II RNA Library Prep Kit for Illumina (New England BioLabs, Ipswich, MA) using the manufacturer's instructions. We had three technical replicates for *B. malayi* and one replicate for the *D. immitis* and *C. elegans* samples. Briefly, RNA samples containing 10 ng of total RNA were fragmented for 15 min at 94°C prior to first and second strand synthesis. The cDNA was purified with 1.8X NEBNext Sample Purification Beads. The cDNA ends were repaired and ligated to Illumina adapters diluted 25-fold. After adapter ligation, the USER enzyme was used to selectively degrade the second cDNA strand, allowing for directional RNA-seq. The libraries were amplified by PCR using NEBNext Multiplex Oligos for Illumina (Dual Index Primers Set 1; New England BioLabs, Ipswich, MA), with a total of 10 cycles. The libraries were purified a final time using 0.9X NEBNext Sample Purification Beads and 1 uL was run on the Bioanalyzer using the High Sensitivity DNA Kit (Agilent, Santa Clara, CA) for validation and quantification. The libraries were pooled and sequenced on a single flow cell of the Illumina NextSeq 550 at a depth of at least 40 million paired-end reads per sample.

Each analysis tool used default options unless otherwise noted. The FASTQ reads were trimmed to remove adapter sequences with the –paired option using Cutadapt (v1.16; Martin, 2011). The quality of each library after trimming was calculated using FastQC (v0.11.9; Andrews, 2023). The trimmed reads were aligned to the genome for each respective species using HISAT2 (v2.1.0; Kim et al., 2019) with the –fr –rna-strandedness RF and -k 30 options. The trimmed reads were mapped to their reference genome, using the *B. malayi*-4.0 (GCA_000002995.5), ICBAS_JMDir_1.0 (GCA_024305405.1), and WBcel235 (GCA_000002985.3) genomes for *B. malayi, D. immitis*, and *C. elegans* reads, respectively. The SAM alignment files were converted to BAM files, keeping only the primary alignments and mapped reads using Samtools view -b -F 260 (v1.15.1; Li et al., 2009). Samtools view was also used to down-sample to 40 million paired reads per library. BamCoverage from deepTools (v3.5.1; Ramírez et al., 2014) was used to create bedGraph files with 50 base pair (bp) bins, skipping all 0 values. Bedtools (v2.29.2; Quinlan and Hall, 2010) merge with the options “-d 1 -c 4 -o sum” was used to combine the bedGraph bins directly beside one another. To identify rRNA regions with a high number of aligned sequencing reads, we kept all genomic regions with >1,000 reads. The regions were aligned to the nucleotide (nt) database using BLASTN (v2.13.0; Altschul et al., 1990; Camacho et al., 2009), to identify high coverage regions that match available rRNA sequences. A bed file was created for each species containing the genomic coordinates for regions that match rRNA sequences with BLAST and any genes that were assigned as rRNA in the reference annotation. The genomic sequences of these regions, representing the putative rRNA sequences, were obtained using the reference genomes and Bedtools getfasta. The *B. malayi* rRNA sequences were used to design custom rRNA depletion probes.

### 2.5 Ribosomal RNA probe design

Putative ribosomal RNA sequences from *B. malayi* and *w*Bm were fed into the NEBNext Custom RNA Depletion Design Tool (https://depletion-design.neb.com), a public tool which utilizes ChipD to select candidate probes at each position based on predicted melting temperature (Dufour et al., 2010). The result is a set of probes which closely tile the reference sequences. The initial probe set (*n* = 2,020) was clustered to collapse highly similar probes using vsearch where pairwise similarity was defined by (*number of matching columns*)/(*the shortest sequence length*; –cluster_fast –iddef 0 –qmask none; Rognes et al., 2016).

Several similarity thresholds (50, 60, and 70%) were evaluated before selecting the probe set clustered at 60% similarity (*n* = 377). This similarity threshold was chosen because it had ample tiling over all rRNA sequences, with the additional advantage of being synthesized in a single oligo pool (max per pool = 384 oligos). Candidate probe sets were aligned back to the putative rRNA sequences with bbmap (v39.06; https://sourceforge.net/projects/bbmap) to assess coverage. Probe efficacy was predicted *in silico* using bbduk to partition undepleted RNA reads by whether they had high k-mer similarity to the probe set or not. The selected probe set had high similarity to 88.69% of RNA reads, compared to 88.73% for the unclustered output. Following the development of this method, the NEBNext Custom RNA Depletion Design Tool was updated to include a clustering option to support the use of the tool with less well-characterized organisms. We ordered an oligo pool from IDT (Coralville, IA) containing the 377 *B. malayi* rRNA depletion probes at a concentration of 50 pmol/oligo. The oligo pool was resuspended in 25 μL of 10 mM Tris, 0.1 mM EDTA, at pH 7.5 (2 μM per probe final concentration) and stored at −20°C until future use.

### 2.6 RNA enrichment and sequencing

The total RNA, described in Section 2.4, was used to make *B. malayi* RNA libraries prepared with various ribosomal depletion methods. We used 10 ng of total RNA as the starting material for each library. One sample was treated with Terminator 5′-Phosphate-Dependant Exonuclease (Biosearch Technologies, Teddington, UK) using the manufacturer's standard procedure with Reaction Buffer A. The sample was incubated at 30°C for 60 min and the reaction was terminated by adding 1 μL of 100 mM EDTA. The sample was purified using the Monarch RNA Cleanup Kit (50 ug; New England BioLabs, Ipswich, MA), with a final elution of 6 μL.

We also tested different probe sets for RNase H based ribosomal depletion. The NEBNext rRNA Depletion Kit v2 for Human/Mouse/Rat in combination with the NEBNext rRNA depletion probes for bacteria (both from New England BioLabs, Ipswich, MA) were used to deplete the rRNA from *B. malayi* total RNA. We diluted 10 ng of RNA in nuclease-free water to final volume of 9 μL. We added 2 μL of the probe hybridization buffer, 2 μL of the human/mouse/rat v2 rRNA depletion solution and 2 μL of the bacteria rRNA depletion solution. The sample was then treated following the manufacturer's instructions. Briefly, the probes were annealed to the rRNA by incubating at 95°C for 2 min followed by a ramp down to 22°C at a rate of 0.1°C per second. The sample was then treated with RNase H for 30 min at 50°C to degrade the RNA in the RNA/DNA hybrids, followed by a DNase I incubation for 30 min at 37°C. The remaining RNA was purified using 1.8X of the NEBNext RNA Sample Purification Beads and eluted in 7 μL of nuclease-free water.

For the third RNA depletion method, we tested our custom *B. malayi* probe design with three technical replicates. We added 2 μL of our custom oligo pool, described in Section 2.5, to 2 μL of Hybridization Buffer from the NEBNext RNA Depletion Core Reagent Set (New England BioLabs, Ipswich, MA) per replicate. The hybridization mix was then added to 10 ng of total *B. malayi* RNA in 11 μL of nuclease-free water. The samples were then treated following manufacturer's instructions and as described above. We also used the custom *B. malayi* probes on *C. elegans* and *D. immitis* total RNA to test efficacy across nematode species. Both of these libraries started with 10 ng of total RNA, described in Section 2.4, and followed the same RNase H protocol described above using the NEBNext RNA Depletion Core Reagent Set.

Five μL from each elution for all three depletion methods, including the *C. elegans* and *D. immitis* samples, were taken into library preparation using the NEBNext Ultra II RNA Library Prep Kit for Illumina, following the same methods described in Section 2.4. All seven depletion libraries were pooled and sequenced using the Illumina NextSeq 550 to a depth of at least 40 million paired-end reads.

### 2.7 RNA-seq analysis

We used default settings for all analysis tools unless noted otherwise. All *B. malayi* RNA-seq libraries, including the 3 total (untreated), Terminator, Human/Mouse/Rat/Bacteria depleted, 3 custom depleted, and a publicly available Poly(A) enriched library (SRR3111490; Chung et al., 2019), were trimmed using Cutadapt (v1.16; Martin, 2011) with the –paired option and aligned to the *B. malayi*-4.0 (GCA_000002995.5) reference genome, containing the *Wolbachia* endosymbiont (*w*Bm) chromosome, using HISAT2 (v2.1.0; Kim et al., 2019) with the “–fr –rna-strandedness RF and -k 30” options. Samtools view (v1.15.1; Li et al., 2009) -b -F 260 was used on each alignment file to convert to a BAM file and remove all non-primary alignments and unmapped reads. As each library has a different number of sequencing reads, the BAM files were subsampled to 40 million reads each by dividing 40,000,000 by the total number of reads and using this value as the downsampling factor with the -s option. To visualize the read alignments, we used bamCoverage from DeepTools (v3.5.1; Ramírez et al., 2014) to make bigwig files from the filtered BAMs with 50 bp bins and normalization using counts per million (CPM). The bigwig files were visualized using IGV (v2.11.9; Thorvaldsdóttir et al., 2013).

RSeQC (v2.6.4.3; Wang et al., 2012) geneBody_coverage.py was used to visualize the distribution of reads across gene bodies to determine if there is a 5′ or 3′ bias with different depletion methods. FeatureCounts (v2.0.1; Liao et al., 2014) was used to count the number of reads over annotated genomic regions, including protein coding, non-coding and ribosomal genes, with the “-s 2 –primary -t gene -g ‘gene_id”' options set. An additional column was added to the count output files, where each gene id was matched to the assigned “gene_biotype” from the annotation file. The sum of the reads assigned to each gene type was calculated and then divided by the total number of reads to quantify the proportion of reads mapped to rRNA, protein coding, ncRNA, or unannotated regions. The feature count files were also used to plot the correlation between the gene counts for the total vs. the custom depletion libraries. Fragments per million (FPM) was calculated for all three replicates for each library type by first taking the sum of all reads in each sample divided by 1 million to obtain the scaling factor, unique to each individual replicate. The counts for each gene were divided by the scaling factor and the average FPM was calculated for the total and custom depletion replicate libraries, separately. The log10 of the average FPMs was taken and visualized using ggplot2 (Valero-Mora, 2010). Correlations between the custom probe library and the Poly(A) or Terminator library were performed using the same methods, without averaging the Poly(A) and Terminator counts, as only one replicate was used.

Samtools view -L was used to identify which reads mapped to the *w*Bm chromosome using bed files for the whole chromosome and for just the *w*Bm rRNA regions. The reads mapping to the *w*Bm rRNA genes were subtracted from the total number of reads mapping to the whole chromosome, where the remaining reads correspond to *Wolbachia* reads mapping to genes of interest.

To analyze the rRNA depletion capacity of our custom probes on different nematode species, the *C. elegans* and *D. immitis* total and custom depletion libraries were compared. The same trimming, alignment, filtering and downsampling methods were used with their respective reference genomes: WBcel235 (GCA_000002985.3) for *C. elegans* and ICBAS_JMDir_1.0 (GCA_024305405.1) for *D. immitis*. BamCoverage was also used to make bigWig files with 50 bp bins and CPM normalization for visualization in the IGV browser. Samtools view -L was used to count reads mapping to the rRNA region bed files described in Section 2.4.

## 3 Results

### 3.1 RNA enrichment methods

**Figure 1:**
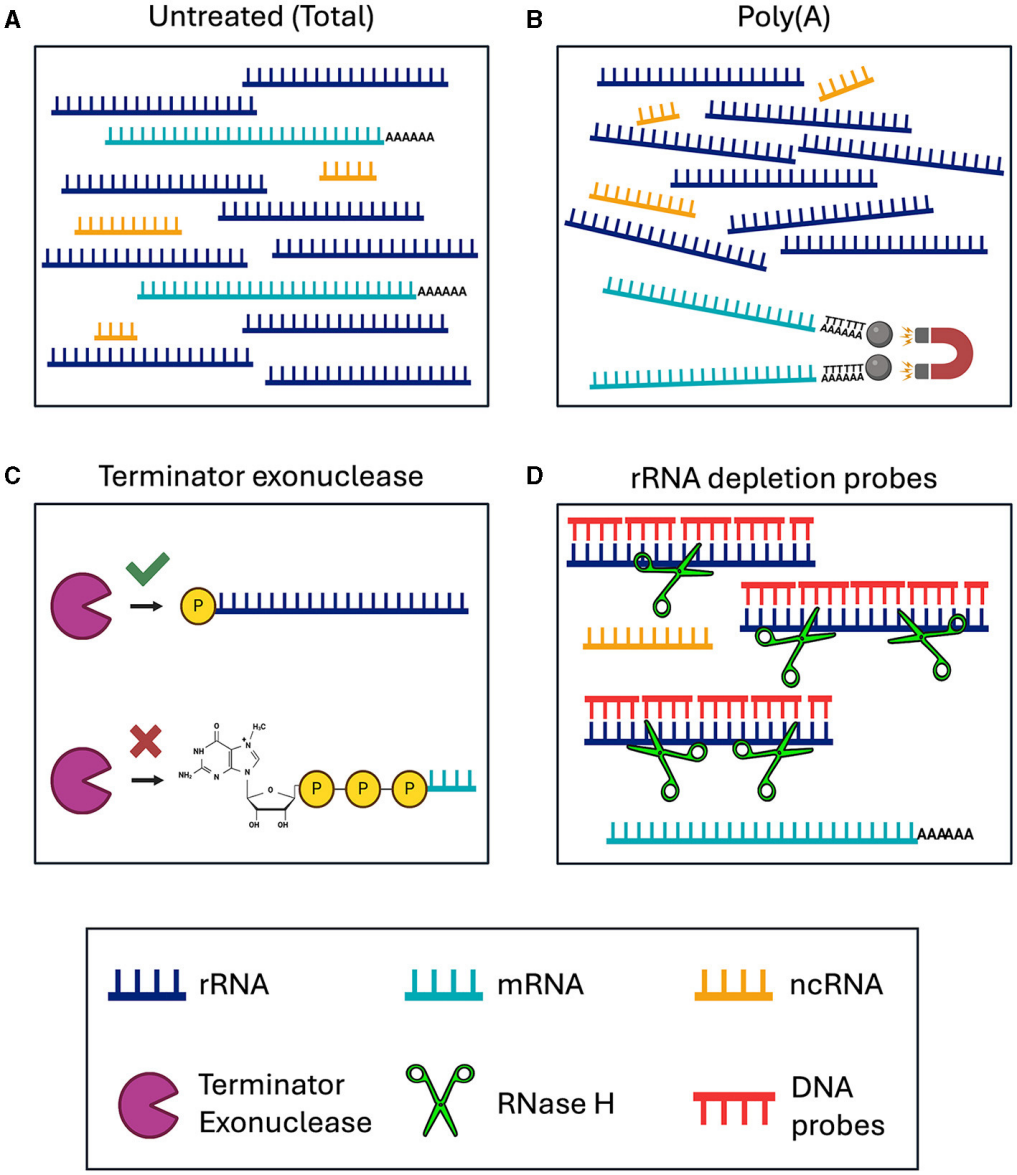
Schematic of RNA enrichment and depletion strategies tested in this study. **(A)** Total RNA sequencing. **(B)** Poly(A) enrichment with magnetic d(T) beads. **(C)** Terminator 5′ Phosphate Dependent Exonuclease treatment. **(D)** RNase H-based ribosomal depletion using sequence specific DNA probes. RNA species are color-coded with rRNA in dark blue, mRNA in teal, and ncRNA in yellow. The Terminator exonuclease is shown in purple, RNase H in green and DNA probes in red.

We tested various RNA-seq enrichment and depletion methods in three nematode species, two of which contain *Wolbachia* as a bacterial endosymbiont. The different library preparation methods should result in sequencing of distinct RNA species (Figure 1, Table 1). Sequencing of *B. malayi* total untreated RNA should result in 80–95% ribosomal sequences, with a small number of mRNA, ncRNA, and bacterial sequences (Figure 1A). This would require large amounts of deep sequencing data to quantitatively study the protein coding gene expression. Poly(A) enrichment is one of the most commonly used methods for eukaryotic RNA-sequencing. This usually involves using Oligo(d)T beads to bind and pull-down the poly(A) tail of certain types of RNA molecules, including mRNA and some types of ncRNA, while removing all rRNA and bacterial RNAs (Figure 1B). However, poly(A) enrichment will remove all bacterial sequences.

**Table 1:** Expected sequencing outcomes from RNA enrichment and depletion methods.

**Library preparation**	**RNA species**	**Bacterial mRNA**
Total (no depletion)	mRNA, ncRNA, and all rRNA	+
Poly(A)	mRNA	-
Terminator exonuclease	mRNA, ncRNA, and 5S rRNA	+
Ribosomal depletion	mRNA and ncRNA	+

The removal of rRNA should enable the user to sequence all ncRNAs and mRNAs, from both eukaryotic and bacteria together in one sequencing library. This can be done using species specific depletion probes or with a phosphate-dependent exonuclease. Here, we tested Terminator 5′-Phosphate Dependent Exonuclease treatment prior to library preparation, which should remove all large rRNA molecules, while retaining mRNA, ncRNA and 5S rRNA, from both the nematodes and *Wolbachia* (Figure 1C). Species specific depletion probes can be designed to remove certain RNA molecules from the total RNA pool based on their nucleotide sequence. Here, we used DNA probes and RNase H to selectively degrade RNA molecules within an RNA/DNA hybrid (Figure 1D). When using DNA probes with sequences complementary to rRNA, we expect to sequence mRNA and ncRNA from both eukaryotes and bacteria. Comparing the sequencing outcomes of these library preparation methods allowed us to determine the most efficient technique for dual RNA-sequencing in samples containing both eukaryotic and prokaryotic RNAs.

### 3.2 Ribosomal depletion in *B. malayi*

We extracted high quality total RNA from *B. malayi* with an RNA Integrity of 9.2 (Supplementary Figure 1A). Two distinct peaks can be seen in the Bioanalyzer trace representing the abundant 18S and 28S rRNAs. After depletion with the custom (Nema) probe set, we observed a significant reduction in the ribosomal peaks (Supplementary Figure 1B). We prepared and sequenced five *B. malayi* RNA-seq libraries using the methods mentioned above. After sequencing, we aligned our reads to the *B. malayi* reference genome (Foster et al., 2020). The read coverage was mapped over annotated genes. No significant 5′ or 3′ bias was observed in any of our libraries, as the quality of the starting total RNA was high (Supplementary Figure 1C), although, the Terminator library has a slight 5′ bias.

**Figure 2:**
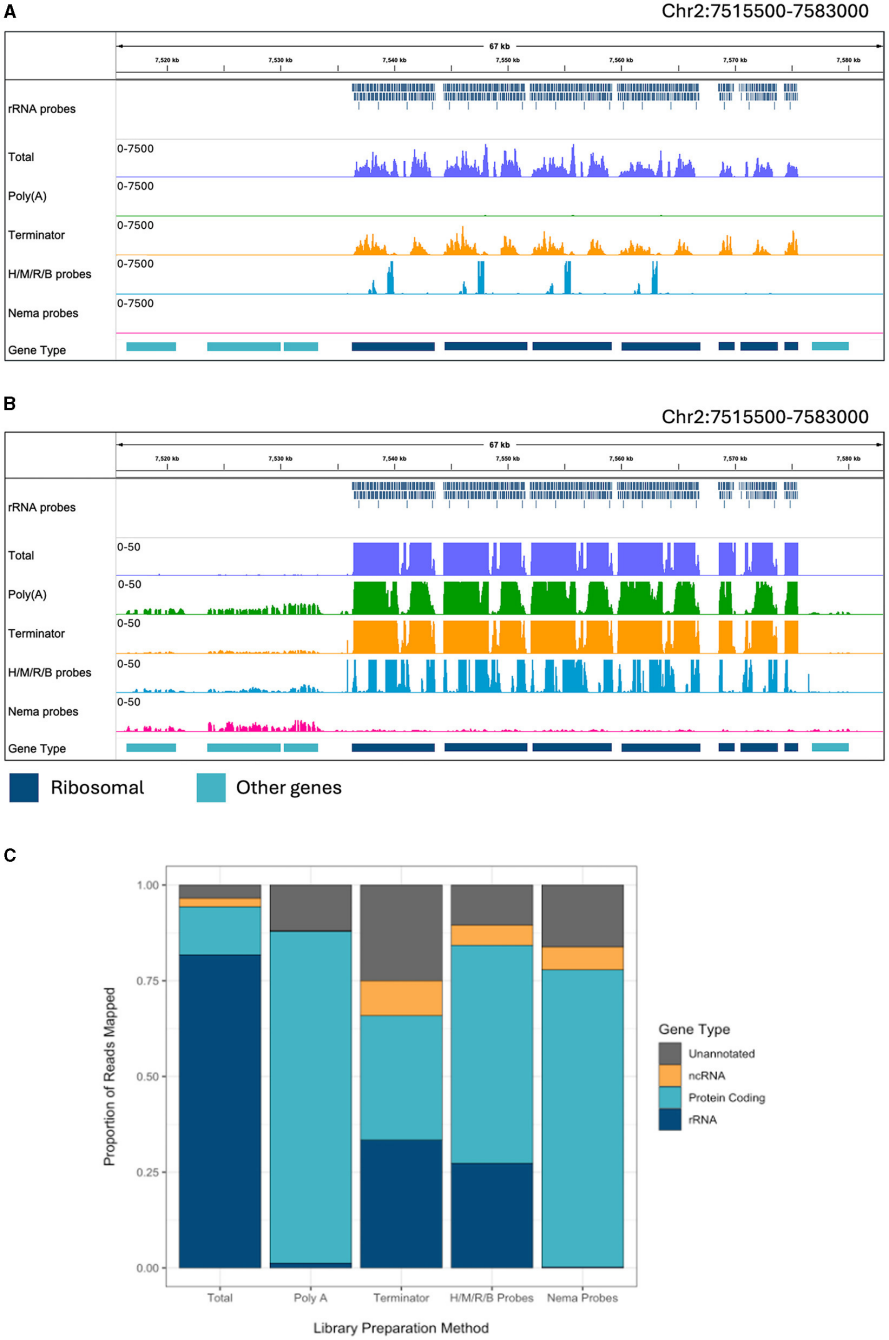
Ribosomal depletion across library preparation methods in *B. malayi*. **(A)** IGV trace showing the custom probes and RNA-seq libraries mapped to the ribosomal tandem repeat region on *B. malayi* Chr2. All BAM coverage files were normalized using CPM in 50 bp bins and shown at a scale of 0–7,500 reads. Protein coding gene annotations are shown in teal and ribosomal gene annotations are shown in dark blue. **(B)** IGV trace showing the same genomic region and coverage files as **(A)** with a scale of 0–50 reads. **(C)** Proportion of reads mapped to each gene annotation type, with unannotated regions in gray, non-coding genes in yellow, protein coding genes in teal. H/M/R/B probes = library made with NEBNext Human/Mouse/Rat rRNA depletion kit combined with the NEBNext Bacteria rRNA depletion probes.

There are a sizeable number of reads mapping to the ribosomal tandem repeat genes in the total RNA sample (Figure 2A). The Terminator library also has a pile up of reads over this region. However, there is a slightly different pattern of read peaks, as some of these regions have been depleted. The NEBNext Human/Mouse/Rat/Bacterial depletion probes remove a large portion of the rRNA reads, leaving 400–600 bp regions undepleted. The Poly(A) and Nema probes both remove almost all of the ribosomal reads. When we observe the read alignments at a smaller scale, we can see reads aligned to protein coding genes surrounding the tandem repeat in every sample besides the total RNA (Figure 2B). However, the Nema probes and Poly(A) libraries have the highest level of mRNA signal. Additionally, we find virtually no reads aligned to the rRNA genes in the Nema probes library, while there appears to be some rRNA carryover in the Poly(A) library.

Each read was assigned to a genomic feature using the *B. malayi* reference genome annotation. We quantified the proportion of reads that map to each feature type for each library preparation method (Figure 2C). All annotated genes, besides protein coding and rRNA genes, were considered non-coding genes. Unannotated reads refer to those mapped to regions that do not contain any known genomic features. RNA-seq experiments often aim to study protein coding (mRNA) or non-coding (ncRNA) gene expression. Around 81% of reads in the total library mapped to rRNA genes, which makes the large majority of the reads unusable for downstream analyses. The Poly(A) library had 1.2% of reads mapping to rRNA and almost no reads mapping to ncRNAs. Unsurprisingly, this results in a substantial increase in mRNA reads. Therefore, Poly(A) enrichment is very useful for studying protein coding genes but will not allow for the study of ncRNAs. The Terminator library still had 33% of reads mapping to rRNA and an increase in reads mapping to unannotated regions, with only 32% of reads mapping to protein coding genes. The Human/Mouse/Rat/Bacteria depletion library had 27% of reads mapping to rRNA genes. While this value is significantly lower than in the total RNA library, there are still a large number of rRNAs that need to be removed when using probes designed for distantly related species. The Nema depletion library had the lowest number of rRNA reads at 0.23%. Notably, this value was even lower than the number of rRNA reads found in the Poly(A) depletion library. In this case, the RNase H method is 5 times more efficient at reducing rRNA carryover than using d(T) magnetic beads. The Nema depletion library also had 6% of reads mapping to ncRNA genes, allowing for the examination of these less studied but functionally important RNA species.

### 3.3 Enrichment of *w*Bm RNA-seq reads after ribosomal depletion

**Figure 3:**
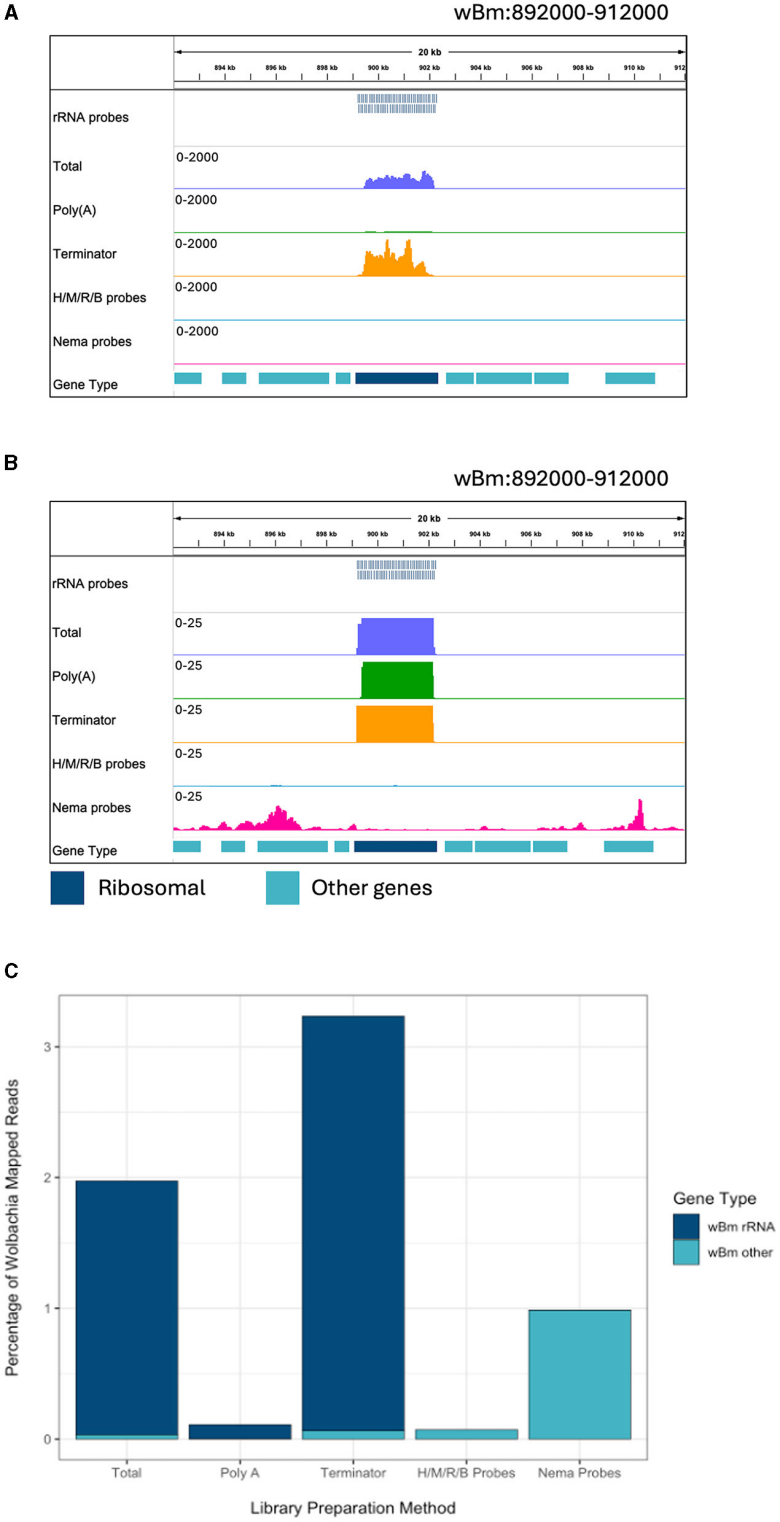
Ribosomal depletion across library preparation methods in *w*Bm. **(A)** IGV trace showing the custom probes and RNA-seq libraries aligned to an rRNA annotated region in the *w*Bm chromosome. All BAM coverage files were normalized using CPM in 50 bp bins. The scale is set to 0–2,000 reads. Protein coding gene annotations are shown in teal and ribosomal gene annotations are shown in dark blue. **(B)** IGV trace showing the same regions and tracks as **(A)**, with a scale of 0–25 reads. **(C)** Percentage of reads mapped to the *w*Bm chromosome from all mapped reads in each library. Reads mapped to rRNA genes are shown in dark blue and reads mapped to all other regions of the *w*Bm chromosome are shown in teal. H/M/R/B probes = library made with NEBNext Human/Mouse/Rat rRNA depletion kit combined with the NEBNext Bacteria rRNA depletion probes.

The Nema depletion probes allow for a more comprehensive analysis of *B. malayi* gene expression. Next, we wanted to determine if this method can also be used to enhance studies of *Wolbachia* gene expression. The *B. malayi* reference genome contains the *w*Bm chromosome. When looking at the reads mapping to the bacteria, we find many reads mapping to rRNA genes in the total and Terminator libraries (Figure 3A). The Terminator enzyme does not appear to have any effective degradation of the bacterial rRNA. When looking at a smaller scale, we observe some rRNA reads present in the Poly(A) library as well (Figure 3B). The Nema depletion library has virtually no reads mapping to the rRNA regions but does contain expression signals in surrounding protein coding genes. Finally, the Human/Mouse/Rat/Bacteria depletion probes do remove the *w*Bm rRNAs, but this does not appear to increase the number of mRNA reads present.

We extracted all reads that mapped to the *w*Bm chromosome from each library. The reads were classified as either rRNA or other *Wolbachia* genes, representing the protein coding and non-coding genes. The proportion of reads mapping to either gene type was compared across libraries (Supplementary Figure 2). The total, Poly(A), and Terminator libraries had over 98% of *Wolbachia* reads mapping to ribosomal genes. Both probe depletion libraries had the opposite proportion, with over 99% of reads mapping outside of the rRNA genes. Next, we determined the percentage of reads mapping to the *w*Bm chromosome out of all the reads within each library (Figure 3C). The total and Terminator libraries had the highest number of *Wolbachia* reads, with around 2 and 3%, respectively. However, these reads do not provide useful information about *Wolbachia* biology as they are primarily rRNA. The Human/Mouse/Rat/Bacteria depletion library had only 0.07% of total reads mapped to *Wolbachia*. The low number of overall *Wolbachia* reads using this method does not allow for accurate quantification of bacterial gene expression. Finally, the Nema depletion library had around 1% of all reads mapping to *Wolbachia*, with almost all of the reads mapping outside of rRNA regions. These data show that the custom filarial nematode depletion probes are the best option for performing rRNA depletion for dual RNA-seq between the worms and their bacterial endosymbiont.

### 3.4 Correlation of gene expression between nema depletion and total RNA-seq

**Figure 4:**
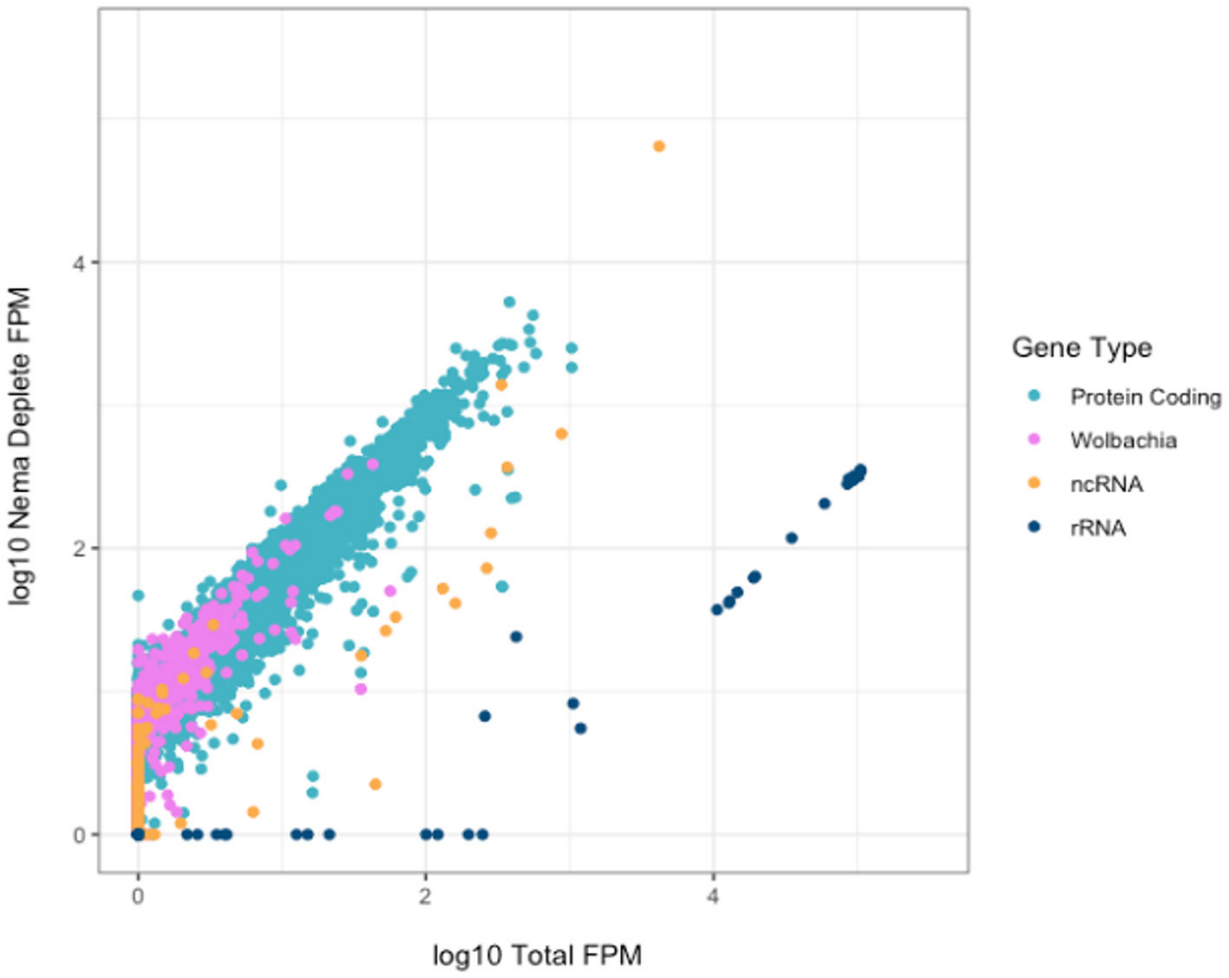
Correlation of gene counts between *B. malayi* Total and Nema depletion libraries. Plot showing the log10 FPM gene counts for the total (x-axis) and custom probe depleted (y-axis) libraries, with rRNA genes in dark blue, protein coding genes in teal, non-coding RNAs in yellow and *Wolbachia* non-rRNA genes in pink.

We investigated the effect of library preparation method on individual gene counts. All gene counts were normalized by calculating the number of fragments per million mapped fragments (FPM). The genes were not normalized for length, as we directly compared the same genes across enrichment and depletion methods. We plotted the Log_10_(FPM) of each annotated gene for the Total and Nema depleted libraries (Figure 4). All gene types, excluding rRNAs, generally had higher read counts in the Nema deplete library. As expected, most rRNA genes had between 100 and 300 times more gene counts in the Total library and no rRNA genes had higher read counts in the Nema depleted sample.

There are some protein coding and non-coding genes that appear to have slightly higher read counts in the Total library. We investigated these genes to ensure that they were not being depleted as a result of off target probe binding. We found no evidence of sequence similarity between these genes and the rRNA probes, as the probe sequences do not map to their genome regions *in silico*. Most genes, such as Bm15518, have the same distribution of reads across the gene body, but with slightly lower coverage in the Nema library (Supplementary Figure 3A). This could be the result of technical artifacts during library preparation and sequencing, as many of these genes had variable gene counts across technical replicates. Bm9361 was the only gene where the distribution of reads differed across the gene body (Supplementary Figure 3B). In the Nema deplete library, a 200 nucleotide sequence around the 3′ end of the gene appears to be depleted. This may represent an off-target effect of the probe set. However, the overwhelming majority of genes have higher gene counts in the Nema deplete library, without any off-target probe binding.

We also compared the Nema depletion library gene counts to those of the Poly(A) and Terminator libraries. The Nema depletion and Poly(A) libraries had similar levels of protein coding gene expression (Supplementary Figure 4A). The Poly(A) library had higher counts in most rRNA genes, almost zero reads in *Wolbachia* genes and minimal reads in most ncRNA genes. The non-coding genes with similar counts in the Poly(A) and Nema depletion libraries probably represent the ncRNAs that contain a poly(A) tail. The Terminator library has higher counts in all rRNA genes, with less enrichment of protein coding and *Wolbachia* genes (Supplementary Figure 4B). There is an enrichment of a small subset of non-coding and protein coding genes in the Terminator library. This could be the result of technical artifacts from sequencing or biological artifacts as a result of their cap or secondary structures.

### 3.5 Ribosomal depletion in other nematode species

**Figure 5:**
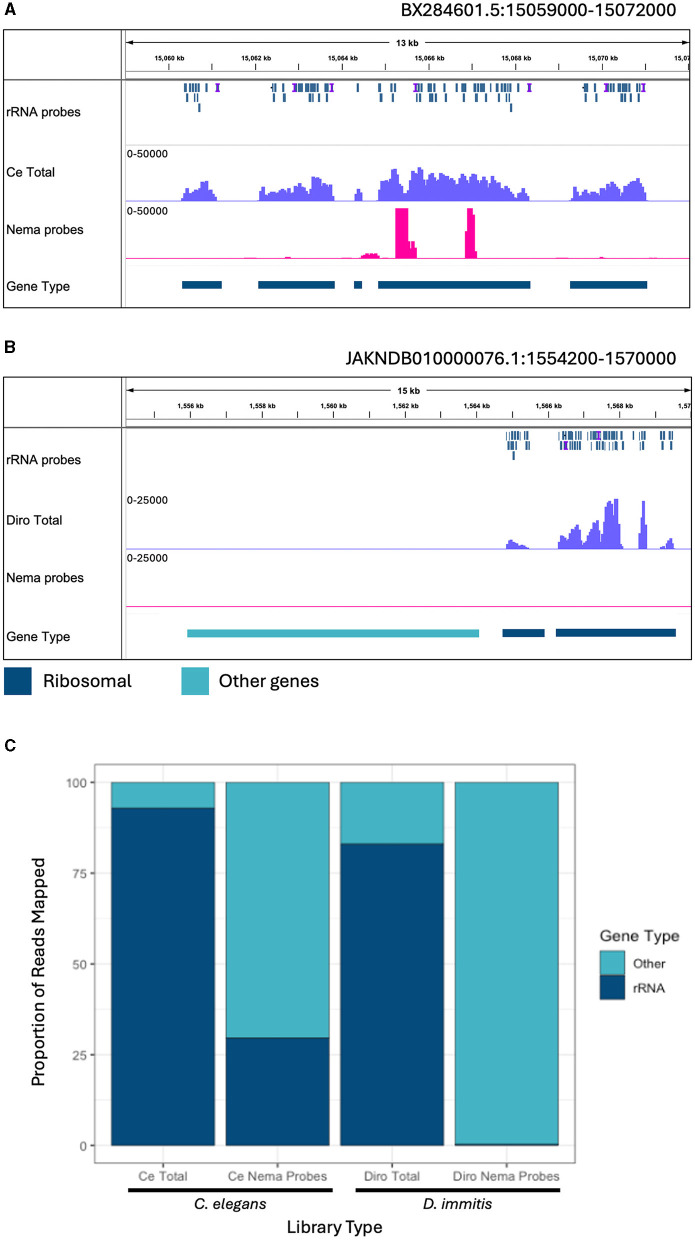
Ribosomal depletion with *B. malayi* custom depletion probes in other nematode species. **(A)** IGV track showing probe mapping in the *C. elegans* genome over ribosomal genes. Additional tracks include BAM coverage files normalized using CPM showing *C. elegans* total RNA (blue) and Nema depletion (pink) libraries at a scale of 0–50,000 reads. **(B)** IGV track showing probe mapping in the *D. immitis* genome over ribosomal genes. Additional tracks show BAM coverage files normalized using CPM of the *D. immitis* total RNA (blue) and Nema depletion (pink) libraries at a scale of 0–25,000. Protein coding gene annotations are shown in teal and rRNA annotations are shown in dark blue. **(C)** Proportion of reads mapping to ribosomal genes vs. other genomic regions in the *C. elegans* and *D. immitis* total and Nema depletion libraries. Reads mapping to ribosomal genes are represented in dark blue. Reads mapping to other genomic regions are shown in teal.

*C. elegans* is a commonly studied model organism in clade V of the nematode phylum (Parkinson et al., 2004). *D. immitis* is a more closely related filarial parasite in the same group, clade III, as *B. malayi*. It is estimated that clades III and V separated over 300 million years ago (Xie et al., 2022). We determined the efficiency of the depletion probes designed with *B. malayi* sequences on these other nematode species. Total and Nema depleted libraries were made with RNA from each species. The libraries were aligned to their species' respective reference genomes, along with the *B. malayi* probe sequences. We observed read and probe alignments over the annotated rRNA genes. In *C. elegans*, the probes have a patchy alignment to the rRNA genes, with many gaps present (Figure 5A). We still see rRNA depletion, however there are two distinct regions, of 500 and 250 bps, where the probes do not align and the rRNA is not depleted. The probes have better alignment to the *D. immitis* rRNA genes and almost all rRNA appears to be removed in the Nema depleted library (Figure 5B). When observing read alignment over protein coding genes, we find an increase in reads for the Nema depleted libraries in both species (Supplementary Figures 5A, B).

We quantified the proportion of reads aligning to rRNA gene annotations from each library (Figure 5C). All reads mapping to ribosomal genes were called rRNA and the remaining mapped reads were classified as other. The *C. elegans* total RNA library had 92.9% of reads mapped to rRNA, with only 7.1% of reads mapped to other genes. The Nema depletion library had 29.5% of reads mapping to rRNA and 70.5% mapped to other regions, indicating that the *B. malayi* probes are not as effective at removing these sequences in this distantly related nematode species. In the *D. immitis* total library, 83% of reads mapped to annotated rRNA genes, with 17% mapping to other genes. The Nema depletion library had only 0.3% of reads mapping to rRNA regions and 99.7% mapping to other regions. The *B. malayi* rRNA probes efficiently removed these sequences at a similar rate across both filarial species. This probe design can most likely work as a pan-filarial nematode ribosomal depletion method.

## 4 Discussion

Enrichment and depletion methods in RNA-sequencing are important for studying biologically interesting gene expression in species across life cycle stages and conditions. Here, we presented ribosomal depletion strategies to study filarial parasites and their endosymbiotic bacteria. We tested poly(A) enrichment, Terminator exonuclease depletion, and species-specific probe depletion, including commercially available and custom probes, on nematode total RNA samples. The custom probes were designed against the *B. malayi* and *w*Bm ribosomal sequences using the NEBNext Custom RNA Depletion design tool, resulting in 377 non-redundant oligo sequences. We obtained complete removal of rRNA using these probes in *B. malayi* samples, which was a significant improvement compared to all other methods tested. There was less rRNA contamination with this RNase H-based method when compared to poly(A) bead enrichment. We found that the probes have negligible off-target effects on other gene sequences. The Terminator enzyme is advertised as a method to remove large rRNA sequences. While we did observe a reduction in the rRNA reads, over 30% of reads still mapped to ribosomal genes.

The Terminator method also appears to enrich and deplete certain protein coding and non-coding genes. Commercially available 5′ Phosphate-dependent exonucleases have been shown to be affected by the secondary structure of RNA. This method also had a slight 5′ sequencing bias when compared to the other library preparation methods (Supplementary Figure 1C). These data show that probe based ribosomal depletion methods should be preferred over current 5′ Phosphate-dependent exonuclease methods.

The NEBNext Human/Mouse/Rat probes combined with the NEBNext Bacteria probes also removed a large proportion of ribosomal reads. However, there were specific regions of these genes that were not removed, leaving 30% of reads in rRNA genes. These regions likely correspond to sequences that have diverged significantly over time between vertebrates and nematodes. The addition of the Bacteria probes did efficiently remove the *Wolbachia* rRNAs.

The custom probes allowed for sequencing of non-coding and bacterial RNAs alongside the protein-coding RNAs. One limitation of this method is the necessity of an annotated reference genome or a total RNA-seq library to identify the abundant sequences. However, the nema depletion was the only method that resulted in abundant levels of *Wolbachia* sequences, not including rRNA reads. Therefore, this method can be used for dual RNA-seq studies in filarial nematodes containing *Wolbachia*. Sequencing both the eukaryotic and prokaryotic sequences in the same library will help to avoid technical artifacts and batch effects that could arise across *B. malayi* and *Wolbachia* libraries that must otherwise be prepared separately, using different methods. Additionally, these probes can be used to enrich for only *Wolbachia* sequences by combining rRNA depletion with poly(A) depletion. The probe-based rRNA depletion can also help to improve reference genome annotations of non-coding genes, as many of these sequences are removed when commonly used poly(A) enrichment is performed. This library preparation method is helpful in situations where the RNA is of low quality. Degraded RNAs are difficult to sequence using either poly(A) enrichment or Terminator depletion, as these methods rely on intact 3′ and 5′ ends, respectively. The probes are designed to bind over all regions of the rRNA, therefore those sequences can be removed even if the whole RNA molecule is fragmented.

The custom *B. malayi* probes did not completely remove rRNAs from the distantly related *C. elegans* samples. A small number of additional probes could be added to the pool to increase efficiency. However, the *B. malayi* probes were able to completely remove rRNAs from *D. immitis* samples. *B. malayi* and *D. immitis* are in distinct groups in the Filarioidea superfamily (Small et al., 2014). Therefore, we expect that these probes can be used as a high-quality ribosomal depletion method across all filarial parasites and facilitate efficient dual RNA-seq of those species harboring the *Wolbachia* endosymbiont.

## Data availability statement

The datasets presented in this study can be found in online repositories. The names of the repository/repositories and accession number(s) can be found at: https://www.ncbi.nlm.nih.gov/, PRJNA1100530.

## Ethics statement

The manuscript presents research on animals that do not require ethical approval for their study.

## Author contributions

LC: Writing – review & editing, Writing – original draft, Visualization, Validation, Supervision, Methodology, Investigation, Formal analysis, Data curation, Conceptualization. VG: Writing – review & editing, Writing – original draft, Visualization, Methodology, Investigation, Formal analysis, Data curation. LB: Writing – review & editing, Writing – original draft, Software, Methodology, Formal analysis, Conceptualization. JF: Writing – review & editing, Writing – original draft, Supervision, Funding acquisition, Conceptualization.
