# Supplementary material for: Dual RNA-seq in filarial nematodes and Wolbachia endosymbionts using RNase H based ribosomal RNA depletion
Source: Front Microbiol. 2024 May 20;15:1418032. doi: 10.3389/fmicb.2024.1418032 (PMC11144916; doi:10.3389/fmicb.2024.1418032)
Supplement: Supplementary file 1 [file Presentation_1.PPTX]

## Slide 1
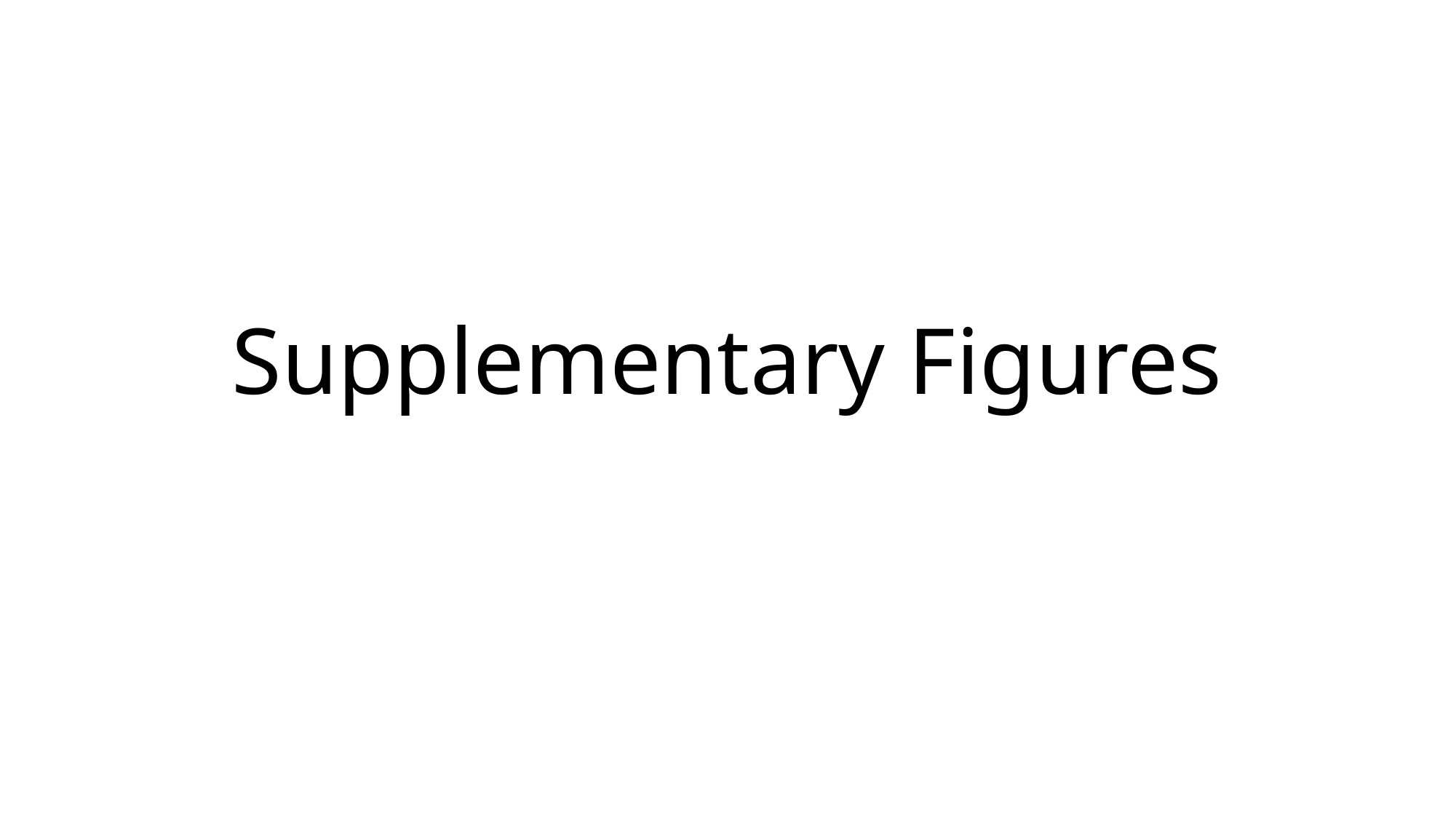

# Supplementary Figures

## Slide 2
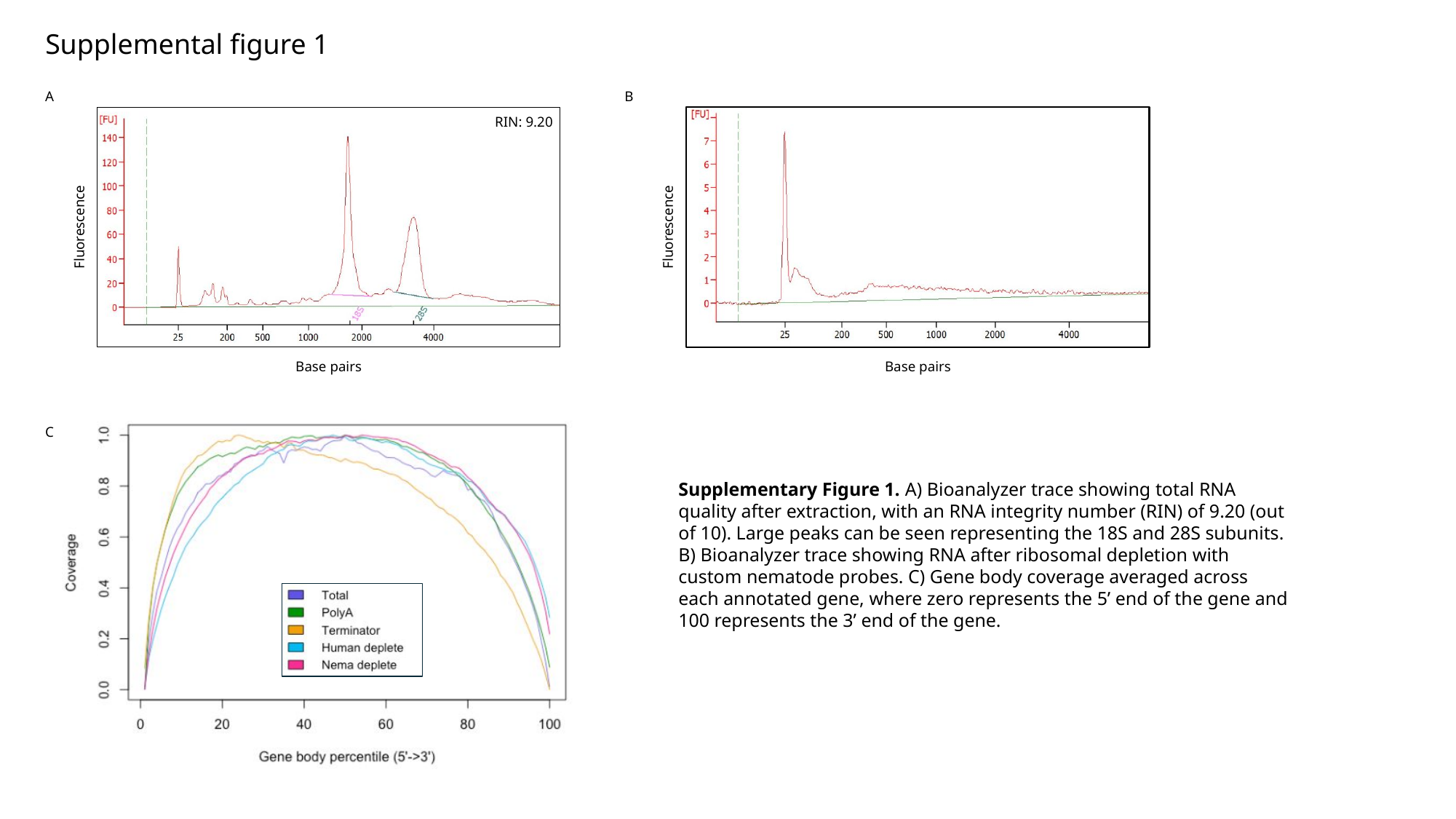

Supplemental figure 1
A
B
RIN: 9.20
Fluorescence
Fluorescence
Base pairs
Base pairs
C
Supplementary Figure 1. A) Bioanalyzer trace showing total RNA quality after extraction, with an RNA integrity number (RIN) of 9.20 (out of 10). Large peaks can be seen representing the 18S and 28S subunits. B) Bioanalyzer trace showing RNA after ribosomal depletion with custom nematode probes. C) Gene body coverage averaged across each annotated gene, where zero represents the 5’ end of the gene and 100 represents the 3’ end of the gene.

## Slide 3
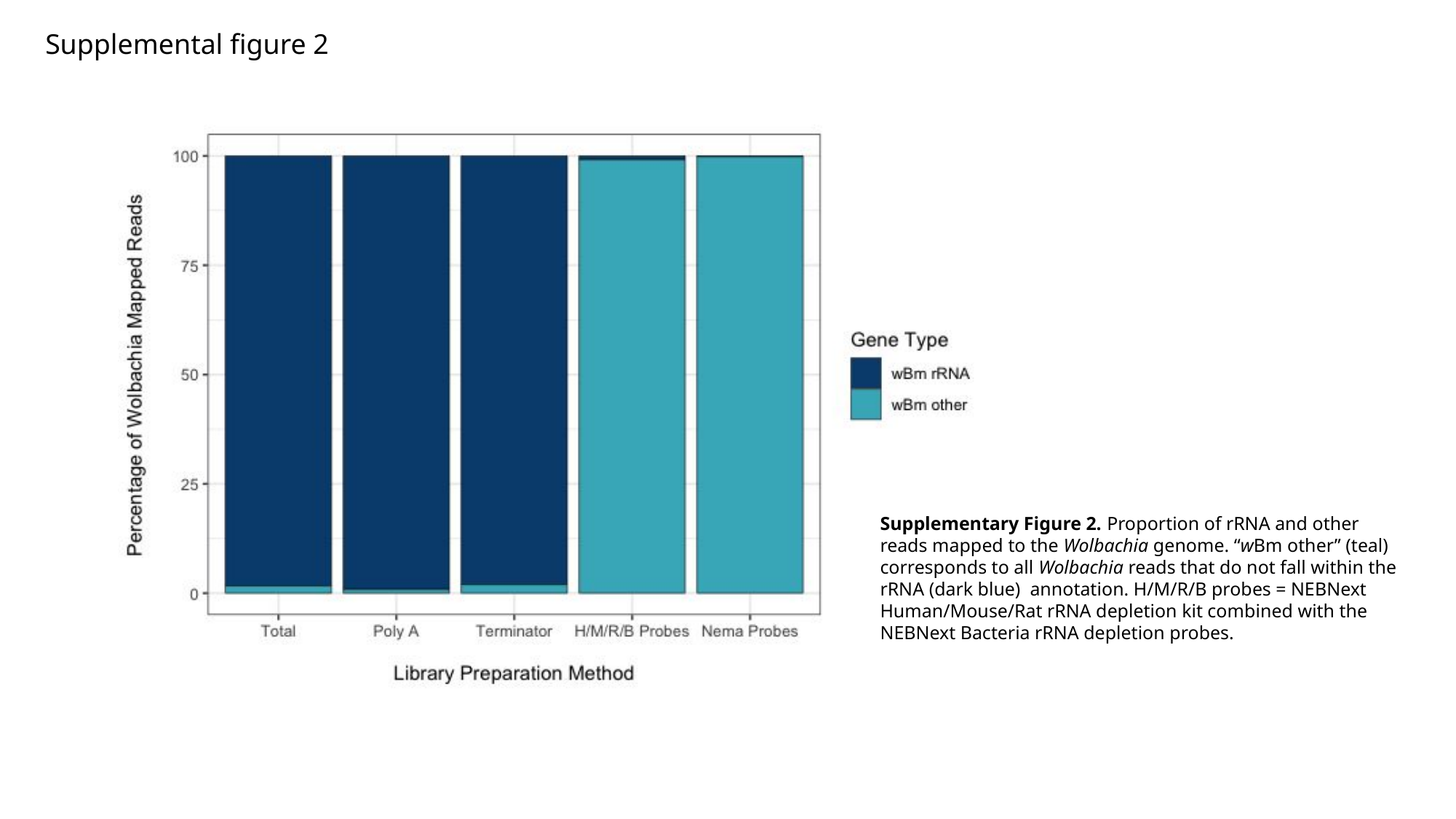

Supplemental figure 2
Supplementary Figure 2. Proportion of rRNA and other reads mapped to the Wolbachia genome. “wBm other” (teal) corresponds to all Wolbachia reads that do not fall within the rRNA (dark blue) annotation. H/M/R/B probes = NEBNext Human/Mouse/Rat rRNA depletion kit combined with the NEBNext Bacteria rRNA depletion probes.

## Slide 4
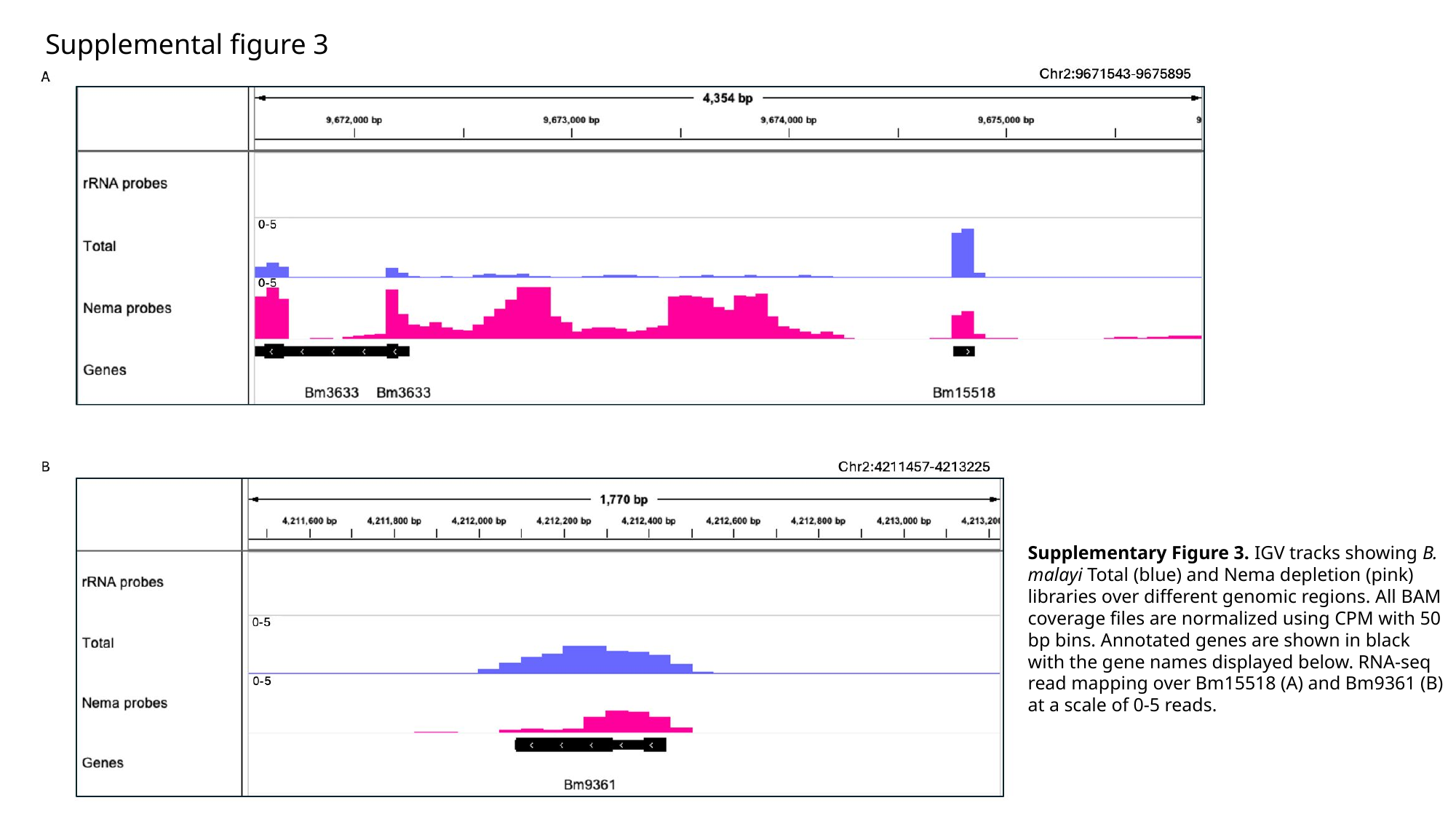

Supplemental figure 3
Supplementary Figure 3. IGV tracks showing B. malayi Total (blue) and Nema depletion (pink) libraries over different genomic regions. All BAM coverage files are normalized using CPM with 50 bp bins. Annotated genes are shown in black with the gene names displayed below. RNA-seq read mapping over Bm15518 (A) and Bm9361 (B) at a scale of 0-5 reads.

## Slide 5
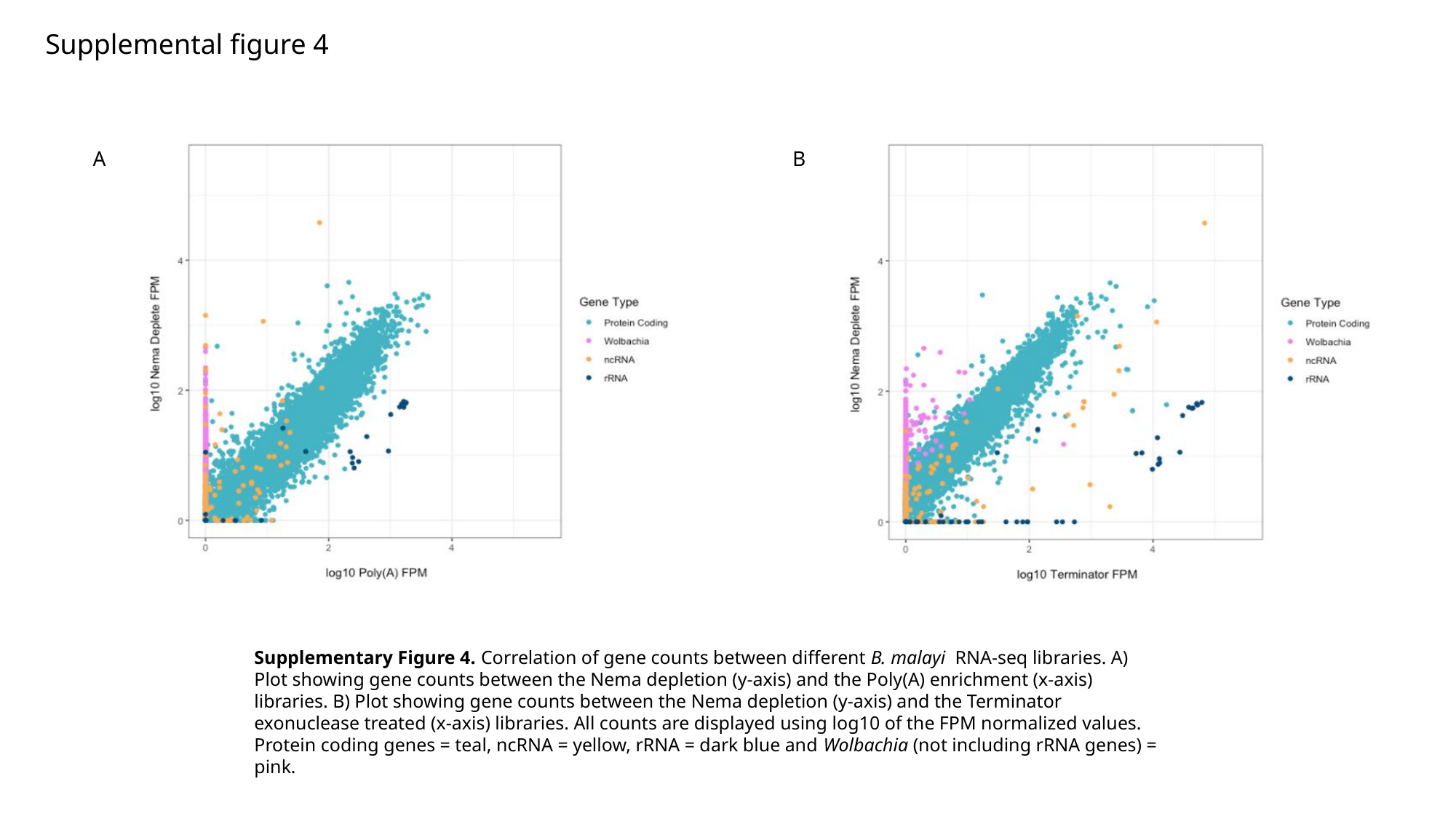

Supplemental figure 4
A
B
Supplementary Figure 4. Correlation of gene counts between different B. malayi RNA-seq libraries. A) Plot showing gene counts between the Nema depletion (y-axis) and the Poly(A) enrichment (x-axis) libraries. B) Plot showing gene counts between the Nema depletion (y-axis) and the Terminator exonuclease treated (x-axis) libraries. All counts are displayed using log10 of the FPM normalized values. Protein coding genes = teal, ncRNA = yellow, rRNA = dark blue and Wolbachia (not including rRNA genes) = pink.

## Slide 6
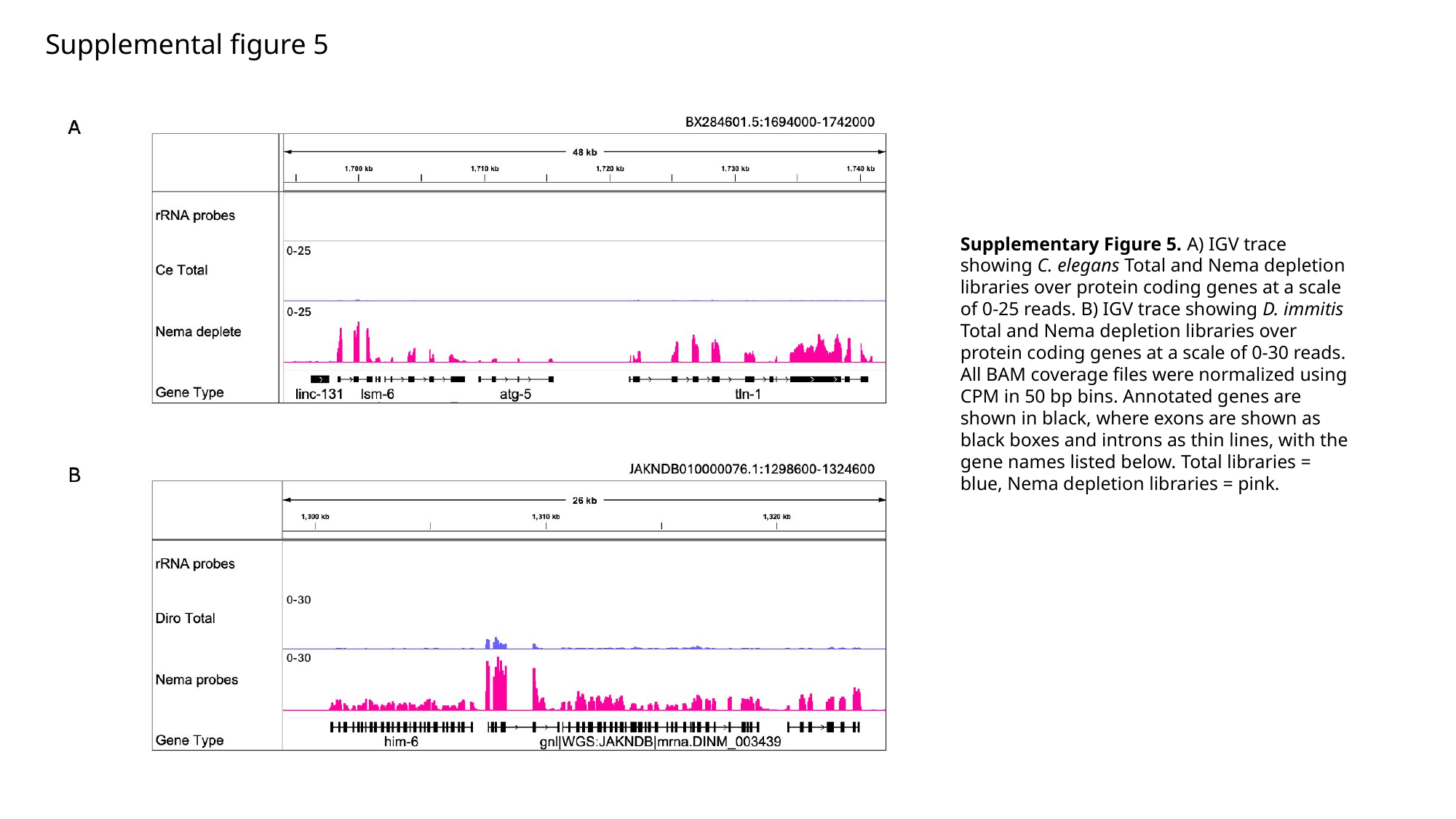

Supplemental figure 5
Supplementary Figure 5. A) IGV trace showing C. elegans Total and Nema depletion libraries over protein coding genes at a scale of 0-25 reads. B) IGV trace showing D. immitis Total and Nema depletion libraries over protein coding genes at a scale of 0-30 reads. All BAM coverage files were normalized using CPM in 50 bp bins. Annotated genes are shown in black, where exons are shown as black boxes and introns as thin lines, with the gene names listed below. Total libraries = blue, Nema depletion libraries = pink.
